# Human Gammaherpesvirus 8 Oncogenes Associated with Kaposi’s Sarcoma

**DOI:** 10.3390/ijms23137203

**Published:** 2022-06-29

**Authors:** Amanda de Oliveira Lopes, Pedro do Nascimento Marinho, Letícia d’Ambrosio de Souza Medeiros, Vanessa Salete de Paula

**Affiliations:** Laboratory of Molecular Virology, Oswaldo Cruz Institute, Oswaldo Cruz Foundation, 4365, Brasil Av., Manguinhos, Rio de Janeiro 21040-360, Brazil; amanda.lopes.fiocruz@gmail.com (A.d.O.L.); marinhopedro@id.uff.br (P.d.N.M.); leticiad@id.uff.br (L.d.d.S.M.)

**Keywords:** Kaposi’s sarcoma, Kaposi’s sarcoma associated herpesvirus, oncogenes

## Abstract

Kaposi’s sarcoma-associated herpesvirus (KSHV), also known as human gammaherpesvirus 8 (HHV-8), contains oncogenes and proteins that modulate various cellular functions, including proliferation, differentiation, survival, and apoptosis, and is integral to KSHV infection and oncogenicity. In this review, we describe the most important KSHV genes [*ORF 73 (LANA)*, *ORF 72 (vCyclin)*, *ORF 71* or *ORFK13 (vFLIP)*, *ORF 74 (vGPCR)*, *ORF 16 (vBcl-2)*, *ORF K2 (vIL-6)*, *ORF K9 (vIRF 1)/ORF K10.5*, *ORF K10.6 (vIRF 3)*, *ORF K1 (K1)*, *ORF K15 (K15)*, and *ORF 36 (vPK)*] that have the potential to induce malignant phenotypic characteristics of Kaposi’s sarcoma. These oncogenes can be explored in prospective studies as future therapeutic targets of Kaposi’s sarcoma.

## 1. Introduction

Kaposi’s sarcoma-associated herpesvirus (KSHV), also known as human gammaherpesvirus 8 (HHV-8), is the causative agent of several malignancies, including Kaposi’s sarcoma (KS), primary effusion lymphoma (PEL), and multicentric Castleman’s disease (MCD) [1]. KS is a multifocal malignancy that mainly affects endothelial cells and is responsible for remarkable morbidity and mortality worldwide, with 34,270 new cases and 15,086 deaths reported in 2020 [2]. In 2020, the highest KS incidence rates were found in countries from Europe (Italy and Portugal), America (Paraguay, Colombia, and Peru), and Africa (such as Mozambique, Zambia, Kanya, Angola, Guinea, Mali, Liberia) Global Cancer Observatory, WHO) [2]. KS is the most common neoplasia in human immunodeficiency virus-infected individuals [3] and in children from endemic regions such as Central, Eastern, and Southern Africa [4]. 

KSHV can establish either latent or lytic infections. The latent phase aims to enhance KSHV persistence in the host by transcription of a limited number of latent transcripts required for episomal maintenance of KSHV in dividing cells and limiting host immune responses [5]. During lytic infection, KSHV replication proteins are transcribed to produce new virions and aid the spread of KSHV to other cells [6,7].

KSHV has a double-stranded linear DNA genome with a length of approximately 165–170 kb. The genome comprises a unique central coding region of approximately 145 kb, and both sides of the region comprise highly GC-rich terminal repeats (TRs) of approximately 30 kb [8,9]. The KSHV genome contains some highly conserved genes from ORFs 4 to 75, which, such as other herpesvirus members, typically encode proteins associated with viral replication and structural virion components. In addition, KSHV encodes a unique set of genes, designated with the prefix K (K1–K15), that have multiple functions in viral infection and virus-induced diseases, a set of viral microRNAs, and several long non-coding RNAs [8].

Several of these genes are viral oncogenes and are host homologs or unique to KSHV. These oncogenes and proteins modulate a variety of cellular functions, including proliferation, differentiation, survival, and apoptosis, and are integral in KSHV infection and oncogenicity [7]. In this review, we show that some of these KSHV genes have the potential to induce malignant phenotypic characteristics of KS. These oncogenes should be explored in prospective studies as future therapeutic targets for KS.

## 2. Kaposi Sarcoma

Kaposi sarcoma (KS) is an angioproliferative neoplasm, firstly described by Moritz Kaposi, an Austro-Hungarian dermatologist in 1872 [10]. KS is typically cutaneous but can involve mucosa or viscera. There are four main epidemiological forms broadly recognized: the first one, originally described by Kaposi, is the classic or sporadic form that affects mostly elderly Mediterranean or Eastern European ancestry men, usually indolent and present almost exclusively on the lower limbs. The second one is the Endemic African form, known for lymphadenopathy occurring in children. The third one emerged from the AIDS epidemic that brought over a particular highly aggressive KS, called Epidemic or AIDS-associated form. KS also has an iatrogenic form, characterized by post organ transplant occurrence, induced by the immunosuppression protocol aiming to reduce organ rejection rates [11].

KS varies from an indolent to an aggressive presentation. Classic and Iatrogenic KS usually present progressive, multicentric, or even ulcerated violaceous, reddish or brownish macules, patches, plaques or nodules on the skin, but possible mucosa involvement following its development stages. Nodular lesions can bleed or become hyperkeratotic as well as polychromatic color change, collarette signs, white lines, among other dermoscopic findings that suggest KS. Usually, these lesions are not invasive and are localized on the lower extremities, with head and trunk, oral or upper limbs possibilities. Iatrogenic KS aggressivity depends on immunosuppression levels and period, being potentially fatal if not regressed [12,13].

Endemic and AIDS-associated KS are more aggressive. Endemic form typically has a lymph node involvement with more diffuse presentation on the body surface, such as the trunk, besides the lower limbs. This form can be divided into four subgroups: nodular, florid, infiltrative, and lymphadenopathic. Nodular African KS is limited to cutaneous lesions. Florid and Infiltrative African KS are aggressive infiltrative local lesions, being deep and extensive cutaneous lesions with bone involvement. Lymphadenopathic African KS is a particular fatal form, affecting children and has a rapid development with viscera involvement [14,15,16]. 

AIDS-associated KS has a predilection for viscera involvement, especially gastrointestinal tract, and pulmonary systems. Usually, oral mucosa is affected, as well as upper limbs, neck and head, besides lower limbs. Due to immunodeficiency in these HIV-patients, AIDS-associated KS has high morbidity and mortality with organ commitment. Patients treated with highly active antiretroviral therapy (HAART) tend to improve immunodeficiency, leading to regression of size and reduction of aggressivity of cancer, resembling a classic KS form [14,15,16].

In men who have sex with men (MSM), the epidemiological and clinical features are quite different from the AIDS-associated KS, resembling more the Classic KS clinic, with particular epidemiology and increasing incidence over the last years, despite the popularization of HIV therapies. There is a proposition of a fifth KS form specifically to this group, called nonepidemic KS [11,17,18].

Staging is not common for Kaposi Sarcoma. However, there is the validated staging classification for only AIDS-associated KS, created in 1980’s pre-HAART era, including the measurement of disease as localized or disseminated (T), immunodeficiency levels by measuring CD4 count as high or low (I) and the presence of systemic symptoms (S), just to classify a poor or good diagnosis. For the other KS forms, there is no universally accepted staging classification [19]. 

Thus, KS negatively influences the quality of life of patients causing many physical and psychosocial problems. Some gastrointestinal KS lesions, for example, may cause pain, bleeding, difficulty with feeding, diarrhea, intestinal obstruction, malabsorption, and weight loss. Pain, severe edema, and cellulitis can also accompany ulcerated skin lesions, and edema can also be present in the absence of skin lesions. Edema of the external genitalia may also obstruct urination. Pulmonary lesions and effusions may be associated with dyspnea, cough, hemoptysis, and restricted activity. Facial and periorbital edema is disfiguring and, in extreme cases, may obstruct vision. Skin lesions, particularly those on the face that are hard to camouflage but also those on the torso and extremities, may lead to self-imposed social isolation, exclusion by others and psychological distress. Many of these problems have most commonly been associated with AIDS-related KS, but they may also occur with all epidemiological forms of the disease [20].

## 3. KSHV Encoded Oncogenes

Oncogenes are genes that have the ability to cause cancer and the activation of these is a major driver of cancer [21,22]. Oncogenes are involved in abnormal cellular-growth control, transformation, and oncogenesis [23]. 

Viral oncogenes play a central role in KSHV infection and oncogenicity cancer [21,22]. During the latency phase, KSHV is able to encode viral oncoproteins, including LANA, vCyclin, and vFLIP. Moreover, during the lytic phase, KSHV encodes viral oncoproteins including vGPCR, vBcl-2, vIL-6, vIRF 1/vIRF 3, K1, K15, and vPK.

### 3.1. Latent Oncogenes

#### 3.1.1. ORF 73 (LANA)

ORF73 measures approximately 4437 kb (Table 1). This oncogene encodes the latency-associated nuclear antigen (LANA) protein, as described in 1997 by Kedes et al. [6,24,25]. LANA, a multifunctional nuclear protein with a length of 1162 amino acids and 220–230 kDa in size, is among the most abundantly expressed proteins during latency. LANA is required for various nuclear functions, including the recruitment of cellular machinery for viral DNA replication and segregation of the replicated genomes to the daughter cells. To carry out this function, LANA binds directly to the conserved TR sequences of the KSHV genome through the protein’s C-terminal domain and docks onto the host chromosome through the N-terminal chromatin-binding domain, which enables the KSHV genome to hitch a ride on the host chromosome during mitosis and maintain a stable copy number in the latently infected cells [5,26].

LANA was found to bind and interact with multiple cellular proteins, including tumor suppressors p53, pRb, and von Hippel Lindau (VHL), transcription factors- ATF4/CREB2 and STAT3, chromatin-binding proteins HP1, H2A/H2B, MeCP2, and Brd4, as well as signal transducer GSK-3β, in order to inhibit apoptosis and stimulate spindle cell proliferation. LANA has also been proposed to bind to several viral promoters and suppress viral lytic gene transcription, thereby influencing latency maintenance. Therefore, LANA is a highly versatile oncogenic protein that plays a central role in the pathogenesis of KSHV [5].

#### 3.1.2. ORF 72 (vCyclin)

In 1996, Chang et al. identified that KSHV ORF 72 encodes a protein, similar to the human cellular D-type cyclin, called KSHV-Cyclin (vCyclin). In 1997, the length and size of the protein (257 amino acids in length and 29 to 30 kDa in size) and a 53% similarity with human D2 cyclins was discovered [27,28]. ORF 72 measures approximately 1.7 kb and is bicistronic, encoded with another latency-associated gene, viral FLICE-inhibitory protein (vFLIP) [6,25,29] (Table 1). 

In human cyclins, vCyclin interacts with cyclin-dependent kinase (CDK)2, CDK4, and CDK6, as well as phosphorylates the cyclin/CDK inhibitory proteins p21 and p27, mediating and inducing the acceleration of the G1/S phase transition. However, unlike D-type human cyclins, whose expression is cell cycle-dependent, the level of vCyclin remains stable throughout the cell cycle and the constitutive activation of vCyclin/CDK complexes in KSHV infected cells appears to result from the extended half-life of vCyclin, which may explain its role in the deregulated proliferation of Kaposi’s sarcoma [30,31].

On the other hand, the ectopic expression of vCyclin in cells with elevated levels of CDK6 has demonstrably proven to lead to apoptotic cell death after the cells enter the S phase, called oncogene-induced senescence [32]. However, this kind of senescence can be relieved by co-expression with vFLIP. In fact, the ratio of vCyclin to vFLIP seems to be important for regulating cell survival and proliferation because vCyclin expression can counteract senescence induced by vFLIP through the activation of the nuclear factor kappa light chain enhancer of activated B cells (NF-κB) [32]. In addition, KSHV-encoded vCyclin can modulate hypoxia-inducible factor 1-α (HIF1α) levels to promote DNA replication during hypoxia [33]. 

#### 3.1.3. ORF 71 or ORFK13 (vFLIP)

ORF 71 (also called K13) measures approximately 566 bp (Table 1). This oncogene encodes vFLIP, a viral homolog of cellular FLICE inhibitory protein, 189 amino acids in length, discovered in 1997 by Thome et al. [6,25,29,34]. FLIPs contain death effector domains that interact with the adapter protein FAS-associated death domain (FADD), inhibiting the recruitment and subsequent activation of the protease FLICE by the CD95 (Fas) death receptor [34].

The best-characterized function of KSHV vFLIP is the utilization of the NF-κB pathway by directly binding to the IκB kinase γ (IKKγ) complex to initiate an extensive range of cellular processes that promote survival, proliferation, differentiation, cytokine secretion, and oncogenic transformation. vFLIP protects cells against growth factor withdrawal-induced apoptosis and plays a very important role in KS infection [35]. vFLIPs from other gamma-herpesviruses were found to protect cells from the Fas protein, tumor necrosis factor receptor (TNFR)-1, translocating chain-association membrane protein (TRAMP), and TNF-related apoptosis-inducing ligand receptor (TRIALR)-mediated apoptosis. KSHV vFLIP protects cells from Fas-mediated apoptosis and permits clonal growth in the presence of the Fas ligand. KS cells were shown to be resistant to Fas-mediated apoptosis before the identification of vFLIP. vFLIP is directly implicated in the pathogenesis of KS because the expression of vFLIP transcripts increases in late-stage KS lesions, which show reduced apoptosis. In addition, vFLIP is likely to be expressed from an internal ribosome entry site (IRES) located within the vCyclin ORF, implicating the relationship in their co-expressing in KS cells and contributing to the emergence and maintenance of this cancer [8,36,37].

### 3.2. Lytic Oncogenes

#### 3.2.1. ORF 74 (vGPCR)

ORF 74 measures approximately 1025 bp (Table 1). This oncogene encodes the viral G protein-coupled receptor (vGPCR) discovered in 1996 by Cesarman et al. [38]. The vGPCR contains 342 amino acids and is a homolog of human interleukin 8 (IL-8) receptors such as CXCR1 and CXCR2 [6,38,39]. This viral oncoprotein, expressed during the lytic cycle, contains seven hydrophobic regions that theoretically correspond to transmembrane domains. The protein also shares other features with members of this class of receptors, including glycosylation sites in the most N-terminal extracellular fragment and two cysteine residues, in the putative second and third extracellular loops, which are conserved in all GPCRs [38].

Although ORF 74 is constitutively active, it is also activated by several human CXC chemokines. Hence, both constitutive and chemokine-induced vGPCR mediated functions may be used by KSHV to promote survival and replication of the virus, while the host immune system also uses the same functions to keep viral survival and replication under control. The transgenic expression of ORF 74 in mice was sufficient for the development of vascular KS-like lesions, indicating that vGPCR plays a critical role in the initiation of KS [40]. The vGPCR-induced onset of Kaposi’s sarcoma involves the stimulation of a complex network of signaling pathways that involve the autocrine and paracrine activation of proliferative, pro-inflammatory, and angiogenic pathways. vGPCR also activates the transcriptional regulator NF-κB, which is involved in the onset of the KS phenotype, including the development of spindle cell-like morphology and the paracrine stimulation of T-cell and monocyte chemotaxis [39].

#### 3.2.2. ORF 16 (vBcl-2)

ORF 16 measures 525 bp [6,41] (Table 1). This oncogene encoded vBcl-2, a viral homolog of the human proto-oncogene Bcl-2, and was described in 1997 by Sarid et al. vBcl-2 is 175 amino acids in length and 19.4 kDa in size [42]. 

Cellular Bcl-2 was originally discovered as an oncogenic protein in B-cell lymphomas. Since then, a number of proteins belonging to the Bcl-2 family have been identified, each possessing the signature Bcl-2 homology (BH) domain. The Bcl-2 family consists of both anti-apoptotic (e.g., Bcl-2, Bcl-X_L_, and Bcl-w) and pro-apoptotic (e.g., Bax, Bak, Bid, and Bad) proteins, which cooperate by forming homodimers or heterodimers to regulate the commitment of cells to apoptosis. Besides Bcl-2, which has the ability to interact with and inhibit pro-apoptotic family members such as Bax and BH3-only proteins, the hydrophobic pocket of Bcl-2 also binds Beclin 1 (the mammalian ortholog of the yeast protein Atg6), which is part of class III PI3 kinase complex, required for the initiation of autophagosome membrane. In addition, the dual roles of Bcl-2 in apoptosis and autophagy suggest that a coordinated regulation may exist for Bcl-2 to conduct these two activities [43].

Given the important role of Bcl-2 in cell survival, many viruses have evolved to encode structural and functional orthologs of Bcl-2 (vBcl-2) to prevent the premature death of infected cells from sustained viral replication and associated diseases [43]. vBcl-2 has the same anti-apoptotic function as cellular Bcl-2. In addition, vBcl-2, in cooperation with Bcl-2, plays a critical role in promoting the development of KS lesions. vBcl-2 interacts directly with cellular proteins to delay apoptosis and autophagy in KSHV-infected cells. Thus, this oncoprotein supports the establishment of KSHV latency and reactivation. However, the regulatory functions of vBcl-2 are not limited to its anti-apoptotic activity [44]. Thus, Liang et al. showed that vBcl-2 plays an important role in KSHV lytic replication, independent of the protein’s anti-apoptotic and anti-autophagic activities [41]. vBcl-2 controls cellular apoptosis and autophagy in the 84WGR86 region, consistent with the findings of previous studies, but controls KSHV lytic replication through the E14 residue, and these functions are genetically separable. Overall, these results identify the novel essential function of vBcl-2 for KSHV lytic replication, which relies on the 14th glutamic acid residue, but not the anti-apoptotic or anti-autophagic activity of vBcl-2 [41]. Other studies showed that the KSHV vBcl2 binds ORF 55 during lytic replication, and this interaction appears to be required for nuclear localization and virion incorporation. In fact, disruption of the vBcl2–ORF 55 interaction by the vBcl2 peptide reduced KSHV virion assembly in the nucleus. As KSHV vBcl2 localizes to the mitochondria and nuclei of infected cells, this vBcl2–ORF55 interaction appears to be necessary for their nuclear translocation [45].

#### 3.2.3. ORF K2 (vIL-6)

ORF K2 measures approximately 676 bp [46] (Table 1). This oncogene encodes the viral interleukin 6 (vIL-6) [47], a viral homolog of the human homolog of interleukin-6 (hIL-6) [48], and was described in 1997 by Neipel et al. [48]. The expression of vIL-6 is highly upregulated during the KSHV lytic phase, but this oncoprotein is also detected during the latent phase [49,50]. vIL-6 has 204 amino acids in length [51] and activates multiple signaling pathways using the hIL-6 receptor complex [49]. 

vIL-6 is an oncoprotein responsible for the development of Kaposi’s sarcoma [51] and induces angiogenesis and hematopoiesis by upregulating VEGF [52]. In addition, the HIV-encoded Nef protein-enhanced vIL-6-induced angiogenesis and tumorigenesis both in vitro and in vivo [53]. Thus, coinfection with HIV remarkably contributes to KSHV infection and further enhances KS tumor pathogenesis.

#### 3.2.4. ORF K9 (vIRF 1)/ORF K10.5 and ORF K10.6 (vIRF 3)

The KSHV genome encodes two viral interferon regulatory factor oncoproteins (vIRF1 and vIRF3) and is a viral homolog of the cellular IRF transcription factor family [54]. ORF K9 measures approximately 1529 bp [46] (Table 1). vIRF 1 is encoded by this oncogenic gene [55] and was described in 1998 by Zimring et al. [56]. This oncoprotein has a length of 449 amino acids [46] and is expressed in both KSHV phases, with low levels during latency and higher levels during lytic replication [57]. 

The ORFs K10.5 and K10.6 measure around 2040 bp [46]. The vIRF 3 is an oncoprotein [55] encoded by these genes, which was described in 1998 by Lubyova et al. [58]. This oncoprotein has 566 amino acids in length [46] and is expressed during KSHV latency [59].

vIRFs are known to interfere with cellular responses to viral infections [60,61]. By disrupting p53 signals, vIRF1 and vIRF3 inhibit apoptosis and induce tumorigenesis in KSHV-infected cells [62]. vIRF3 deregulates IFNs and cell death [63] and can also inhibit apoptosis in the PKR pathway [55].

#### 3.2.5. ORF K1 (K1)

ORF K1 measures approximately 1080 bp [46] (Table 1). The K1 transmembrane protein is an oncoprotein [64] encoded by this gene, described in 1998 by Lee et al. [65]. K1 is 279 amino acids in length [46] and is primarily expressed during KSHV lytic replication [66].

The K1 protein has multiple roles in cellular signal transduction, viral reactivation, endothelial cell immortalization, and host immune recognition [67]. K1 has been reported to bind the c subunit of 5′adenosine monophosphate-activated protein kinase (AMPKc1), which is important for K1 to enhance cell survival [68]. K1 demonstrated the ability to transform rodent fibroblasts [65] and immortalize primary human umbilical vein endothelial cells in vitro [69]. In addition, K1 transgenic mice develop spindle cell sarcomatoid tumors and plasmablastic lymphoma, thought to be mediated by the activation of NF-κB and the B-cell transcription factor, Oct2 [70,71]. K1 is thought to be involved in the activation of tyrosine immunoreceptors that also activate a cascade linked to the tumorigenesis of KS [64].

#### 3.2.6. ORF K15 (K15)

ORF K15 measures approximately 6245 bp [72] (Table 1). This oncogene was described in 1998 by Nicholas et al. [73] and encoded the K15 protein, with approximately 488 amino acids in length [46].

K15 is a transmembrane viral protein with structural similarity of LMP2A of the virus Epstein Barr, and like LMP2A, K15 can block BCR signaling [72,74]. K15 activates the mitogen-activated protein kinase (MAPK) pathway, NF-kB pathway, and PLCc1 through tumor necrosis factor receptor-associated factor 2 (TRAF2)’s interaction with its SH2-binding site leading to the expression of proangiogenic and proinflammatory factors [75,76,77]. K15 has also been shown to interact with the anti-apoptotic protein HAX1 and induce the expression of several anti-apoptotic genes, which can provide a survival advantage to the infected cell [75,78]. K15 has been shown to be important for viral lytic replication and this protein is expressed in a substantial proportion of KSHV-infected endothelial spindle cells in KS lesions [75].

#### 3.2.7. ORF 36 (vPK)

ORF 36 measures approximately 2900 bp [46] (Table 1) and was firstly described in 2000 by Park et al. [79]. This oncogene encodes a serine/threonine viral protein kinase (vPK) and, although there are differences in location and function, vPK has homology with other herpesviruses kinases [79,80,81]. Protein kinases phosphorylate cellular proteins and alter substrate localization, enzymatic activity, and protein interactions, affecting signal transduction and global cellular function [82]. vPK has 444 amino acids of length [46] and can enhance host cellular protein synthesis by phosphorylating ribosomal protein S6, and to promote of cell replications [81,83]. The vPK protein mimics S6KB1 protein, phosphorylating S6 [82], and the acetyltransferase TIP60, a regulator of chromatin remodeling. The DNA damage response is phosphorylated by this protein, activating it and promoting cell replication [81]. vPK can also be expressed by hypoxic environments [82]. In fact, Anders et al. demonstrated that viral protein kinase could promote B cell activation and proliferation and augment lymphomagenesis in vivo [81]. Therefore, vPK contributes to the development of KSHV cancers, including KS. KS biopsies from KS-afflicted patients show detectable vPK transcripts [81,82,84].

## 4. Challenges of Current KS Therapies and Potential KSHV Viral Oncogene as Therapeutics Targets

Standard Kaposi’s sarcoma therapy has not changed in the last years and as KS manifests in many forms, therapies should also be divided into multiple application scenarios [85]. Thus, the therapeutic management is based on an individual approach analyzing the criteria of disease extension, the localized or disseminated character of the lesions, predictors of disease evolution, immunovirological status of the patients, and patient’s comorbidities [19]. Different therapies can be used for KS, such as immune restoration, radiotherapy, and chemotherapy [85]. 

Several therapies have been developed for localized lesions but without randomized trial comparisons. Radiotherapy is one of the most efficient treatments for this KS type. However, higher doses per fractions and concomitant administration of systemic therapies should be avoided to reduce the risk of sequelae. The possible risks are out-of-field recurrence, and radiotherapy-induced skin toxicity (telangiectasia, hyperpigmentation, skin atrophy, and fibrosis) [19]. Surgical excision has a high recurrence rate and can cause severe functional impairment [19,86]. CO_2_-laser and superficial cryotherapy can be temporarily efficient in superficial lesions [19,86,87]. Intralesional chemotherapies have good response rates. Vinblastine (the most used) [88] and vincristine [89] are examples of this therapy type [19]. Electrochemotherapy combines intralesional chemotherapy, usually bleomycin, with electroporation, enhancing drug uptake into tumoral cells and response rates. Topical treatments-imiquimod [90] or topical 9-cis-retinoid acid (alitretinoin gel 0.1%) [91] can also be used [19].

Systemic treatments are performed for locally aggressive extensive and disseminated KS. The most recommended therapies are pegylated liposomal doxorubicin (PLD) and paclitaxel (PCT) chemotherapies [19]. The PLD safety profile is good, with around 5% grade IV neutropenia and 5% hand and feet syndrome [19,92,93]. PCT has more grade toxicity compared to PDL, particularly more grade IV neutropenia and mild-to-moderate alopecia [19,94]. Other chemotherapies, such as vinblastine [95], etoposide [96,97], and bleomycin [98], can be considered as therapy alternatives but are not used/recommended as first-line therapies [19]. 

In classic KS, PLD or low-dose interferon-alfa is the recommended first-line agents in younger patients. In AIDS KS, combination antiretroviral therapy is the first treatment option, but specific systemic treatment is recommended in case of extensive disease. Systemic treatment is also used in the prevention and treatment of immune reconstitution inflammatory syndrome in these patients [19]. PLD is approved as first-line therapy, and PCT is approved as the second-line for AIDS KS [19,94,99]. In post-transplant KS, tapering down immunosuppressive therapy and switching to mammalian target of rapamycin (m-TOR) inhibitors are used [19].

The use of KSHV oncoproteins as therapeutic targets has been gradually becoming more in focus as a novel strategy to treat and prevent the KS lesions growth. However, these studies are preliminary [75,100,101,102,103,104,105,106].

**Figure 1:**
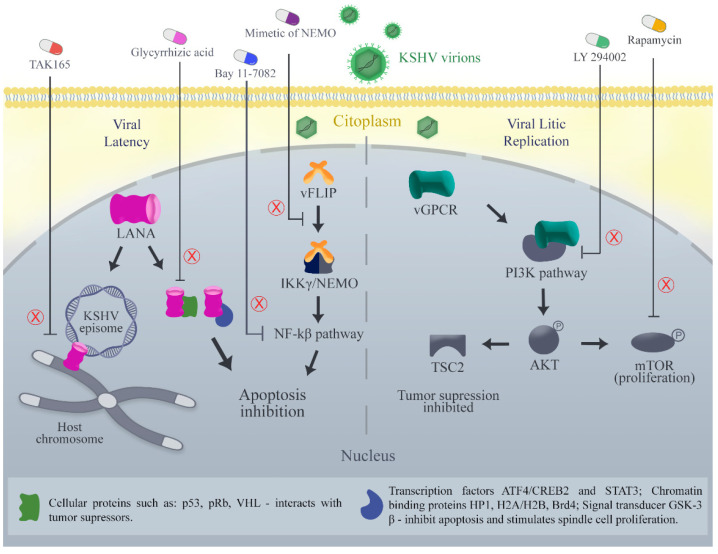
Therapeutics targets in signaling pathways modulated by Kaposi sarcoma-associated virus (KSHV) oncoproteins. The pathways shown include PI3K/Akt/mTOR associated with vGPCR (lytic oncoprotein), NF-kB associated with vFLIP (latency oncoprotein), and different pathways associated with LANA (latency oncoprotein). A total of 5 therapies are represented outside the cell and their respective targets are indicated with the letter “X” in red color.

LANA binds in the terminal repeat region of the KSHV genome and, after this, docks onto the host chromosome, which ensures the viral genome replication and segregation during cell mitosis [5,26] (Figure 1). Kirsch and colleagues discovered and optimized new small compounds able to inhibit the binding of LANA to KSHV DNA in the low micromolar range [107,108,109,110]. In addition, Mubritinib (TAK165), protein kinase inhibitor, was identified as a potent inhibitor of LANA-DNA binding and strongly reduced living KSHV PEL cells in vitro and in vivo [110,111] (Figure 1). The decreasing LANA expression in PEL cells with shRNA [112], by treatment with glycyrrhizic acid [113] (Figure 1) or HSP90 inhibitors [114] induces cell death [110]. 

Another potential target is the vFLIP, a potent activator of the NF-kB pathway and neutralizer apoptosis [35] (Figure 1). Silencing vFLIP using NF-kB inhibitors such as Bay 11-7082, induces PEL cell apoptosis [110,115,116,117,118]. In order to activate the NF-kB pathway, vFLIP directly interacts with IKKγ/NEMO. A tertiary protein structure mimic of the vFLIP interaction site in the IKKγ/NEMO helix was able to induce cell death in PEL cells and to delay tumor growth in a PEL xenograft mouse model [110,118] (Figure 1). Additionally, a conformationally constrained, stapled IKKγ peptide derived from the IKKγ–vFLIP interaction site interferes with the binding of IKKγ to vFLIP and enhances apoptosis in PEL cell lines [119]. Thus, it may be feasible to develop small molecule inhibitors targeting the vFLIP-IKKγ/NEMO interaction [110]. 

vGPCR develops angioproliferative tumors in multiple organs, such as KS. This tumorigenesis is mediated through numerous pathways, the most important of which appears to be the phosphatidylinositol 3-kinase (PI3K) pathway ([100,120,121,122,123,124]) (Figure 1). PI3K is a lipid kinase that activates Akt, a serine-threonine kinase that has multiple targets, including the mammalian target of rapamycin (mTOR), a kinase that is associated with cell proliferation and survival in KS ([100,124,125]). In vitro, cells expressing constitutively active vGPCR have high levels of activated Akt, inactivated TSC2 (a tumor suppressor which is inactivated by Akt), and activated mTOR ([124,126,127]). This has been reversed with LY 294002, a PI3K inhibitor, or rapamycin, an mTOR inhibitor, in vitro and in vivo murine models; the latter was also associated with decreased tumor growth ([124,127]) (Figure 1).

Other therapies and their respective therapeutic targets in signaling pathways modulated by KSHV oncoproteins, such as vIL-6 [128], have been described. Oroxylin A inhibits vIL-6-mediated lymphatic reprogramming of vascular endothelial cells through modulating PPARγ/Prox1 axis. Thus, this therapy may serve as a candidate for the treatment of KS [128].

## 5. Conclusions

KSHV encodes many viral oncogenes. The information on KSHV oncogenes associated with Kaposi’s sarcoma reported here has provided a basis for molecular studies of KS tumorigenesis. The development of inhibitors of proteins associated with these oncogenes would be a novel possibility for anticancer therapy of Kaposi’s sarcoma.

## Figures and Tables

**Table 1:** KSHV oncogenes involved in KS conditions.

	Oncogenes KSHV										
	ORF 73	ORF 72	ORF 71/ORF K13	ORF 74	ORF 16	ORF K2	ORF K9	ORF K10.5/K10.6	ORF K1	ORF K15	ORF 36
***First description***	Kedes et al., 1997	Chang et al., 1996	Thome et al., 1997	Cesarman et al., 1996	Sarid et al., 1997	Neipel et al., 1997	Zimring et al., 1998	Lubyova et al., 1998	Lee et al., 1998	Nicholas et al., 1998	Park et al., 2000
***Genomic location***	Latency-associated region	Latency-associated region	Latency-associated region	Lytic replication associated region	Lytic replication associated region	Lytic replication associated region	Lytic replication associated region	Lytic replication associated region	Lytic replication associated region	Lytic replication associated region	Lytic replication associated region
***Length (~bp)***	4437	1700	566	1025	525	676	1529	2040	1080	6245	2900
***Oncoprotein***	LANA	vCyclin	vFLIP	vGPCR	vBcl-2	vIL-6	vIRF 1	vIRF 3	K1	K15	vPK
***Function***	Viral DNA replication and segregation of the replicated genomes to the daughter cells; inhibition apoptosis; KS cell proliferation	Acceleration of the G1/S phase transition; KS cell proliferation	Survival, proliferation and cell differentiation; cytokine secretion; oncogenic transformation; inhibition of apoptosis	Viral survival and replication; activation of pro-inflammatory, and angiogenic pathways	Inhibition of apoptosis and autophagy; viral replication	Induction angiogenesis and hematopoiesis	Inhibition of apoptosis and induction tumorigenesis	Deregulation cellular responses to viral infections; inhibition of apoptosis; induction tumorigenesis; deregulation cell death	Cellular signal transduction; viral reactivation; endothelial cell immortalization; host immune recognition; activation of tyrosine immunoreceptors	Viral lytic replication; inhibition of apoptosis; activation pro-inflammatory and angiogenic pathways	Cell proliferation and induction angiogenesis

Note: bp, base pairs.

## Data Availability

Not applicable.
